# Cyclosporine (0.05%) Combined with Diclofenac Sodium Eye Drops for the Treatment of Dry Eye Disease

**DOI:** 10.1155/2022/2334077

**Published:** 2022-10-15

**Authors:** Run Bai, Li-ping Liu, Zhen Chen, Qiong Ma

**Affiliations:** Department of Ophthalmology, Urumqi Military Region General Hospital Urumqi Xinjiang, Urumqi 830099, China

## Abstract

**Objective:**

To assess the clinical efficacy of cyclosporine (0.05%) combined with diclofenac sodium eye drops (0.1%) in the treatment of dry eye disease.

**Methods:**

A prospective analysis was performed on clinical information of 128 patients diagnosed with dry eye at the ophthalmic clinic of the General Hospital of Xinjiang Military Command of the Chinese People's Liberation Army from August 2020 to August 2021. Specifically, patients were randomly divided into a control group and a study group. In addition to conventional treatment, patients in the control group were treated with cyclosporine (0.05%) eye drops; while in the study group, patients received cyclosporine (0.05%) combined with diclofenac sodium eye drops (0.1%). Subsequently, comparisons and analysis were performed before and after treatment between the two groups in the clinical symptom questionnaire score of dry eye disease, the corneal fluorescein staining (CFS) score, determination of tear film break-up time (BUT), Schirmer I test (SIT) score, and curative effect.

**Results:**

After treatment, the clinical symptom scores and CFS scores were decreased while the BUT and SIT scores were increased in both groups; besides, compared with the control group, the clinical symptom scores and CFS scores were much lower while the BUT and SIT scores were higher in the study group. Moreover, the overall response rate in the study group (96.9%) was much better than that in the control group (79.7%); and the differences between the two groups were statistically significant (*p* < 0.05).

**Conclusion:**

The combination of cyclosporine (0.05%) and diclofenac sodium eye drops (0.1%) based on conventional treatment can be applied to the clinical treatment of dry eye disease due to its good clinical effects on relieving dry eye symptoms.

## 1. Introduction

Dry eye disease, a general term for a variety of diseases accompanied by ocular discomfort and/or ocular tissue damage, is mainly presented as abnormalities in tear quality or quantity due to any cause or decreased tear film stability due to abnormal dynamics [1]. In 2007, the Report of the TFOS International Dry Eye Workshop published by the Tear Film and Ocular Surface Society (TFOS) proposed the definition of dry eye. Briefly, dry eye is a multifactorial disease of the tear and ocular surface characterized by eye discomfort, defects of vision, and a loss of homeostasis of the tear film. Besides, dry eye is usually accompanied by ocular symptoms like increased tear osmolality and ocular surface inflammation, which may damage the ocular surface [2]. Approximately 14% of adults in the United States suffer from symptomatic dry eye; moreover, the morbidity is higher in women and increasing with age [3]. Notably, the total social expenditures related to dry eye disease, including treatment management, physician visits, productivity losses, and others, exceed $55 billion annually [4]. There are various causes of dry eye disease, such as eye surgery, autoimmune diseases, excessive usage of visual display terminals, excessive use of eyes at work, wearing corneal contact lenses, and terrible eye habits [5]. Mainly clinical treatments for dry eyes include fumigation, hot compress, lacrimal punctum embolization, moisture chamber glasses, gland transplantation, artificial tear substitutes, and corticosteroid eye drops [6]. However, these treatments have many side effects because they tend to increase tear film volume by reducing tear drainage or supplementing aqueous solutions. In addition, the abovementioned treatments do not target the underlying etiologies, so they fail to cure dry eye disease fundamentally [6]. Therefore, exploring effective treatments for dry eye disease is crucial to relieve the clinical symptoms in patients.

Recently, inflammation has been considered as an important mechanism for the pathogenesis and progression of dry eye disease [7], and as a result, anti-inflammation has emerged as a significant treatment. Cyclosporine, an anti-inflammatory drug with immunomodulatory effects, has been approved to treat dry eye disease by the U. S. Food and Drug Administration [8]. However, cyclosporine is mainly soluble in oil-based emulsions due to its high hydrophobicity; these emulsions are poorly tolerated, have a short residence time on the ocular surface, and accompany by a high irritation [8]. Additionally, long-term use of cyclosporine may cause side effects such as nephrotoxicity, neurotoxicity, hypertension, and hyperlipidemia [9]. Therefore, developing novel drugs with good efficacy and minimal side effects is urgent for the clinical treatments of patients with dry eye disease.

Nonsteroidal anti-inflammatory drugs can inhibit cyclooxygenase (COX) and block the synthesis of prostaglandins (PGs, modulators of inflammation), thereby attenuating inflammation [10]. Studies have reported good efficacy of nonsteroidal anti-inflammatory drugs in treating dry eye disease [11]. Diclofenac sodium (2-((2,6-dichlorophenyl) amino) sodium phenylacetate) is a commonly applied nonsteroidal anti-inflammatory drug. Liu et al. stated that diclofenac sodium eye drops combined with sodium hyaluronate relieved the clinical symptoms of patients with dry eye disease [12]. Li et al. claimed that patients receiving diclofenac sodium eye drops combined with artificial tear gel had a considerably higher cure rate, a normal rate of tear secretion, and a normal tear rate than those in the control group [13]. However, the therapeutic effect of cyclosporine combined with diclofenac sodium eye drops on dry eye disease has not been reported yet, and it is also unclear whether the combination can effectively treat dry eye disease and improve clinical symptoms.

Therefore, the objective of this study was to explore the clinical efficacy of cyclosporine combined with diclofenac sodium eye drops on dry eye disease. Briefly speaking, we prospectively analyzed the clinical information of 128 patients diagnosed with dry eye disease at the ophthalmic clinic of the General Hospital of Xinjiang Military Command of Chinese People's Liberation Army. After treatment with the combination of cyclosporine (0.05%) and diclofenac sodium eye drops, the changes in ocular symptoms and physical signs scores of patients were compared. Overall, this study provided a theoretical basis for the clinical treatment options of patients with dry eye disease.

## 2. Subjects and Methods

### 2.1. Study Subjects

This study was approved by the Ethics Committee of the General Hospital of Xinjiang Military Command of Chinese People's Liberation Army (approval number: 2022-061). In this study, 128 patients diagnosed with dry eye disease in the ophthalmic clinic of the General Hospital of Xinjiang Military Command of Chinese People's Liberation Army from August 2020 to August 2021 were selected. The patients composed of 76 females and 52 males aged from 36 to 78 (mean 57 ± 5.5) years. They were then equally and randomly divided into a control group (*n* = 64) and a study group (*n* = 64), with 38 females and 26 males in each group.

### 2.2. Inclusion Criteria

Patients with dry eye disease were identified according to the clinical symptoms, signs, and the accessory examination results of the inclusion criteria [14, 15]. In brief, clinical symptoms included dryness, foreign body sensation, grinding, photophobia, burning sensation, asthenopia, red eyes, itching, and blurred vision; patients with four or more of the abovementioned symptoms were enrolled in this study. Clinical signs included the presence of conjunctiva congestion, follicular hyperplasia in palpebral conjunctiva, tears debris or abnormal mucous secretions in the conjunctival sac, infiltration of punctate (or filamentous), or tear stains in the corneal epithelium; patients with two or more of the abovementioned signs were enrolled in this paper. In addition, accessory examinations composed of tear film break-up time (BUT) test (≤10 s) or Schirmer I test (SIT; ≤10 mm/5 min) and corneal fluorescein staining (CFS; ≥2 points). All in all, patients diagnosed with dry eye based on the abovementioned criteria were included in this study.

### 2.3. Exclusion Criteria

Patients were excluded if they presented with (1) heart, brain, lung, liver, and renal dysfunction; (2) a history of ocular trauma and ocular surgery; (3) meibomian gland dysfunction; (4) Sjögren's syndrome and long-term use of glucocorticoids; and (5) poor compliance. Besides, pregnant and lactating women were also not allowed to be involved in this study [16, 17].

### 2.4. Treatment Regimen

Both groups were treated with conventional therapy (cleansing of abnormal secretions from the surface of the cornea, hot compresses, or/and heat fumigation, etc.). Specifically, patients in the control group were treated with cyclosporine (0.05%) eye drops (GYZZ H20203239, Shenyang Xingqi Pharmaceutical Co Ltd.), with 1-2 drops/time, 2 times/d in both eyes, and 12 hour interval required. As for the study group, patients were given cyclosporine (0.05%) combined with diclofenac sodium eye drops (0.1%) (GYZZ H10960176, Shenyang Xingqi Pharmaceutical Co Ltd), with 1-2 drops/time and 3-4 times/d in both eyes; and the application of both eye drops was separated by 3-5 min/time. Drug uses could be increased or reduced based on individual differences or their symptoms and signs. Notably, patients with severe dry eye disease (SIT ≤3 mm/5 min, BUT ≤3 s, and corneal damage in two or more quadrants observed by CFS inspection) were treated 4-6 times/d. By the way, the treatment was given with 14 d as a course, for a total of 14-60 d [18, 19].

### 2.5. Outcome Measures

After different treatments, the clinical symptoms of patients with dry eye disease in the two groups were evaluated by questionnaires. In addition, the CFS, TBUT, and SIT indexes were also measured and statistically analyzed.

#### 2.5.1. Clinical Symptom Questionnaire of Dry Eye Disease

The dry eye questionnaire designed by Zhao et al. has high reliability, specificity, and sensitivity, is in line with Chinese eye use habits, and has better diagnostic value in the clinical diagnosis of dry eye disease than that of the Ocular Surface Disease Index (OSDI) questionnaire [20]. In brief, the contents of the questionnaire included subjective symptoms (the presence of eye dryness, foreign body sensation, grinding, photophobia, burning sensation, asthenopia, red eyes, itching, blurred vision, etc.), the options (no, occasional, frequent, and persistent), and corresponding scores (0, 1, 2, and 3).

#### 2.5.2. Corneal Fluorescein Staining (CFS) Assay

After seating, patients were asked to look upward, and fluorescein sodium ophthalmic strips (Liaoning Meizilin Pharmaceutical Co Ltd, China) were placed in the conjunctival sac of the lower eyelid. Subsequently, the patients were asked to close their eyes for a few seconds to distribute the fluorescein evenly over the surface of the eyeball, and then, the corneas of the patients were observed by a slit lamp. On basis of the 12-point method, the corneas were divided into 4 quadrants, each quadrant for 0-3 points. Briefly, 0 points represented staining; 1 point was equal to 1-30 punctate stains; 2 points meant >30 punctate stains but without diffusing; and 3 points indicated diffuse stains, filamentous infiltration, and ulcer on the cornea [21].

#### 2.5.3. Tear Film Break-Up Time (BUT)Assay

The patients were required to take a seat and look upward, and then fluorescein sodium (0.05%) was instilled into their lower conjunctival sac. After blinking several times, the patients were allowed to open their eyes, and their corneas were observed under the slit lamp. Finally, the time difference between the last blink and the presence of the first black spot on the corneal surface was recorded by using a stopwatch, and repeated measurements were performed three times to take the mean value. Additionally, BUT >10 s was classified as normal, 5 s < BUT ≤ 10 s as mild, 3 s < BUT ≤ 5 s as moderate, and 0 s < BUT ≤ 3 s as severe.

#### 2.5.4. Schirmer I Test (SIT)

A 5 mm × 35 mm Schirmer tear test strip (Tianjin Jingming New Technological Development Co Ltd, China) was folded 5 mm at one end and then placed at 1/3 outside of the lower conjunctival sac (without topical anesthesia). The patients took a seat and closed their eyes naturally. After 5 min, the strip was removed, and the length of the moist strip was measured from the fold. Specifically, SIT ≥ 10 mm/5 min was regarded as normal, 5 mm/5 min ≤ SIT < 10 mm/5 min as mild, 3 mm/5 min ≤ SIT < 5 mm/5 min as moderate, and SIT < 3 mm/5 min as severe [22].

### 2.6. Efficacy Evaluation

The comparison of the severity of the disease before and after treatment was considered as the efficacy scoring criteria. The specific efficacy criteria are shown as follows: (1) cure: clinical symptoms and signs disappeared during reexamination; BUT > 10 s, CFS < 1/3 corneal area, and SIT of multiple measurements >10 mm/5 min; (2) excellently effective: clinical symptoms and signs were subsided; the condition observed by the slit lamp was greatly improved; the fraction of sum scores of symptoms and signs during reexamination was ≥80%; 7 s ≤ BUT < 10 s, 1/3 ≤ CFS < 2/3 corneal area, and SIT of multiple measurements ≥5 mm/5 min; (3) effective: clinical symptoms and signs as well as the condition observed by the slit lamp were improved; the fraction of sum scores of symptoms and signs during reexamination <80%; 4 s ≤ BUT < 7 s, 1/2 < CFS ≤ 2/3 corneal area, and multiple SIT of multiple measurements <5 mm/5 min; (4) ineffective: the fraction of sum scores of symptoms and signs during reexamination was unchanged or increased, and related indexes did not reach the corresponding criteria.

### 2.7. Statistical Analysis

Statistical analysis was performed using the SPSS v26.0 software. Measurement data were expressed as mean ± standard deviation (SD), and a *t*-test was adopted for comparison among groups. Count data were expressed as (%), and *χ*^2^ test or F test was used for the comparisons between the two groups. A value of *p* < 0.05 indicated a statistically significant difference.

## 3. Results

### 3.1. Comparison of the General Data of Patients between the Two Groups

**Figure 1:**
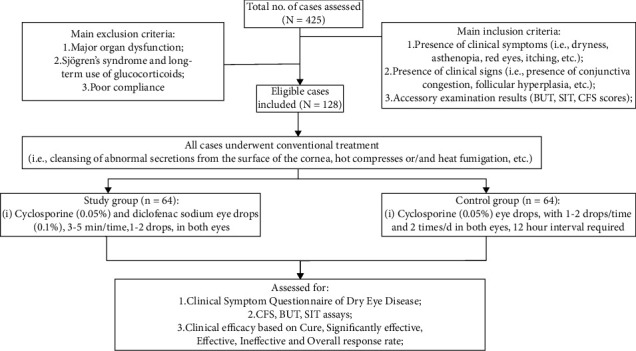
Flow chart to show the study population and design.

A total of 128 patients with dry eye disease were enrolled in this study. Based on the same gender ratio, patients were equally assigned to a control group (*n* = 64) and a study group (*n* = 64), consisting of 38 females and 26 males in each group. There was no statistical difference in general data such as gender, age, and disease duration (*p* > 0.05), indicating the comparability between the two groups. A flow chart of the study population and design is shown in Figure 1.

### 3.2. Comparison of Observation Indicators between the Two Groups

Before treatment, there was no significant difference in clinical symptom scores in questionnaires of dry eye disease, CFS, BUT, and SIT scores between the two groups (all *p* > 0.05). After treatment, the clinical symptom scores and CFS scores in both groups were decreased (control) while the BUT and SIT scores were increased (*p* < 0.01) compared with those before treatment. Additionally, after treatment, the clinical symptom (3.8 ± 0.47 *vs.* 5.7 ± 0.57, *p* < 0.001) and CFS scores (1.2 ± 0.23 *vs.* 4.1 ± 0.49, *p* < 0.001) of the study group were much lower while the BUT (9.0 ± 0.67 *vs.* 5.5 ± 1.10, *p* < 0.001) and SIT (6.9 ± 1.73 *vs.* 3.2 ± 1.43, *p* < 0.001) scores were higher than those of the control group (Table 1). In a nutshell, on the basis of conventional treatment, the efficacy of cyclosporine (0.05%) combined with diclofenac sodium eye drops on dry eye disease was preferred to cyclosporine (0.05%) eye drops alone.

### 3.3. Comparison of Clinical Efficacy between the Two Groups

Subsequently, the clinical efficacy of the two groups was evaluated (Table 2). The results showed that the percentage of patients cured in the study group was higher than that in the control group (35.9% *vs.* 21.9%). Furthermore, the overall response rate was 96.9% in the study group while 79.7% in the control group, and the difference between the two group was statistically significant (*χ*^2^ = 12.86, *p* < 0.05). The abovementioned finding proved that the response rate of cyclosporine (0.05%) combined with diclofenac sodium eye drops to dry eye disease was significantly higher than that of cyclosporine 0.05% eye drops.

## 4. Discussion

Currently, there are very few studies on the efficacy of cyclosporine and diclofenac sodium regimen in the treatment of dry eye, and most of them are limited to animal experiments. Kilic and Kulualp used 56 female mice that were induced dry eye syndrome to compare the therapeutic efficacy of formal saline, sodium hyaluronate, diclofenac sodium, olopatadine, retinoic acid, fluorometholone, cyclosporine A, and doxycycline hyclate based on the assessment of blink rate, tear production, tear BUT, and impression cytology prior to (baseline) and for a period of 2, 4, and 6 weeks [23]. Overall, they concluded that diclofenac sodium and cyclosporine A were most effective in treating dry eye syndrome in the mouse models; besides, these agents exerted therapeutic effects via their anti-inflammatory activities. The study of Kilic et al. was one of the considerations that made us perform this paper. In this article, a large cohort of 128 patients with dry eye disease were given conventional treatment, followed by different treatments. On basis of observation results, the combination of cyclosporine (0.05%) and diclofenac sodium eye drops was more effective than cyclosporine (0.05%) eye drops only.

Considering that the main pathogenesis of dry eye disease is inflammation, common anti-inflammatory drugs such as steroids have been used for clinical practice; however, long-term use of such drugs may induce serious adverse events [24]. The mechanism of action of steroids is by inhibiting the COX activity. Actually, nonsteroidal anti-inflammatory drugs can prevent the synthesis PGs. To be specific, COX, as an important enzyme protein in the progression of inflammation, can catalyze arachidonic acid to produce inflammatory mediators such as PGs and tumor necrosis factors (TNFs), thereby accelerating inflammatory progression [25]. In addition, due to the lack of steroid rings, nonsteroidal anti-inflammatory drugs are strongly safe and do not develop side effects of steroidal hormones, such as ocular hypertension, stromal keratitis, and inhibition of epithelial cell growth [26]. Furthermore, El-Shazly et al. claimed that ketorolac and nimesulide were the nonsteroidal anti-inflammatory drugs that could improve tear volume, SIT, and TBUT scores in the dry eye model of albino rabbits [25]. Aragona et al. reported that nonsteroidal anti-inflammatory drugs like indometacin and diclofenac improved corneal sensitivity and ocular surface damage in patients with Sjögren's syndrome [27]. Likewise, as a nonsteroidal anti-inflammatory drug, bromfenac sodium can improve the symptoms of patients with dry eye disease who are poorly treated with artificial tears [11].

Diclofenac sodium, the nonsteroidal anti-inflammatory drug employed in this study, is frequently used and has a high safety profile in the treatment of ocular inflammatory diseases such as keratitis, conjunctivitis, and postoperative inflammation. Moreover, diclofenac sodium has no adverse effects similar to those observed in the application of cyclosporine or steroid hormones [28]. Moreover, related clinical studies have also revealed ameliorative effects of diclofenac on patients with dry eye disease [12, 13]. Based on this paper, we believe that diclofenac sodium exerted its therapeutic efficacy by decreasing the synthesis of endogenous PGs and inhibiting COX [29].

Cyclosporine is an immunosuppressive agent which can significantly inhibit the formation of antibodies and cell-regulated immune responses, protect nerves, and treat allergic inflammation [30]. Cyclosporine eye drops are widely applied to treat dry eye disease because they can maintain the health status of the ocular surface by reducing the infiltration of lymphocytes in the lacrimal glands and conjunctival tissue, downregulating the expression of inflammatory factors, and promoting the increase of the mucin secretion and the generation of the protective film over the ocular surface [31]. Nevertheless, cyclosporine is largely soluble in oily excipients, and these excipients bring great irritations to eyes; besides, prolonged usage of cyclosporine is prone to cause side effects [8, 9]. Some reports have proved that cyclosporine combined with nonsteroidal anti-inflammatory drugs improves symptoms more quickly than cyclosporine alone in patients with severe dry eye disease [32]. In our study, the combination of cyclosporine and diclofenac sodium was applied to treat dry eye disease. The results showed that the combination treatment significantly relieved the clinical symptoms of dry eye disease. Shortly speaking, the clinical symptom questionnaire and CFS scores were significantly lower while the TBUT and SIT scores were higher in the study group than in the control group. What's more, the overall response rate of the study group (96.9%) was markedly higher than that of the control group (79.7%). All in all, the efficacy of the combined treatment was better than that of cyclosporine alone.

In this study, two patients in the study group did not respond to treatments due to the presence of numerous tear punctate and filamentous infiltrates on the cornea, severe conditions, long disease duration, irregular early medication, and their poor compliance with the treatment. Given the complex etiology of dry eye disease, appropriate treatment should be selected to improve the ocular surface according to the signs of patients. Collectively, cyclosporine combined with diclofenac sodium eye drops exhibits good efficacy and can serve as an effective treatment regimen for dry eye disease. In fact, the combination treatment can synergistically strengthen the therapeutic effect and obviously relieve the clinical symptoms of dry eye disease.

Despite interesting findings reported in this study, there were some limitations worth mentioning. Firstly, we did not observe patients' adverse events during treatment, so whether the combination regimen could reduce the adverse events associated with long-term application of the drug alone still requires further investigation. Secondly, the sample size in the study was small, and long-term follow-up was not performed. In another word, the effectiveness and safety of the combination treatment still need to be validated by a multicenter prospective analysis based on a large sample size and long-term follow-up.

## 5. Conclusion

Overall, on the basis of conventional treatment, cyclosporine (0.05%) combined with diclofenac sodium eye drops effectively relieves the clinical dry eye symptoms and increases response rates. In short, the combination of cyclosporine (0.05%) and diclofenac sodium eye drops shows good clinical efficacy and promising applicability for the clinical treatment of patients with dry eye disease.

## Figures and Tables

**Table 1:** Comparison of clinical symptoms, CFS, BUT, and SIT scores between the two groups.

Variable	Clinical symptom score		CFS score		BUT score		SIT (mm/5 min)	
	Before treatment	After treatment	Before treatment	After treatment	Before treatment	After treatment	Before treatment	After treatment
Study group	8.1 ± 0.79	3.8 ± 0.47^*∗∗*^	7.3 ± 0.14	1.2 ± 0.23^*∗∗*^	4.0 ± 0.29	9.0 ± 0.67^*∗∗*^	2.5 ± 1.20	6.9 ± 1.73^*∗∗*^
Control group	8.0 ± 0.67	5.7 ± 0.57^*∗∗*^	7.3 ± 0.30	4.1 ± 0.49^*∗∗*^	4.1 ± 0.30	5.5 ± 1.10^*∗∗*^	2.2 ± 1.05	3.2 ± 1.43^*∗∗*^
*t*	0.757	−20.387	−0.075	−42.653	−1.774	21.820	1.505	13.177
*p*	0.451	<0.001	0.940	<0.001	0.078	<0.001	0.135	<0.001

Note: NFS, corneal fluorescein staining; BUT, break-up time; SIT, Schirmer I test; ^*∗∗*^*p* < 0.01*vs*., before treatment.

**Table 2:** Comparison of clinical efficacy between the two groups.

Group	Case	Cure, *n* (%)	Significantly effective, *n* (%)	Effective, *n* (%)	Ineffective, *n* (%)	Overall response rate (%)
Study group, *n* (%)	64	23 (35.9)	25 (39.1)	14 (21.9)	2 (3.1)	96.9
Control group, *n* (%)	64	14 (21.9)	19 (29.7)	18 (28.1)	13 (20.3)	79.7

## Data Availability

The data used to support the findings of this study are available from the corresponding author upon request.
